# Triage of Limited Versus Extensive Disease on ^18^F-FDG PET/CT Scan in Small Cell lung Cancer

**DOI:** 10.22038/aojnmb.2017.8751

**Published:** 2017

**Authors:** Riaz Saima, Bashir Humayun, Niazi Imran Khalid

**Affiliations:** 1 Department of Nuclear Medicine, Shaukat Khanum Memorial Cancer Hospital and Research Centre, Lahore, Pakistan; 2 Department of Radiology, Shaukat Khanum Memorial Cancer Hospital and Research Centre, Lahore, Pakistan

**Keywords:** ^18^F FDG PET-CT scan, Disease free survival, Small cell lung cancer

## Abstract

**Objective(s):**

Small cell lung cancer (SCLC) is an aggressive neuroendocrine carcinoma, which accounts for 10-15% of pulmonary cancers and exhibits early metastatic spread. This study aimed to determine the added value of ^18^F-FDG PET/CT imaging in tumor, node, and metastasis (TNM) staging of SCLC, compared to the conventional computed tomography (CT) scan and its potential role as a prognosticator.

**Methods:**

This retrospective review was conducted on 23 patients, who were histopathologically diagnosed to have SCLC and referred for undergoing ^18^F-FDG PET/CT scanning during October 2009-December 2015. The rate of agreement between the CT and ^18^F-FDG PET/CT findings for TNM staging was calculated using the Cohen’s kappa (κ). The median follow-up time was eight months, ranging 27-3 months). The overall and disease-free survival rates were calculated based on the extent of disease.

**Results:**

19 cases were male and four female with the mean age of 58±9 years. The ^18^F-FDG PET/CT identified limited and extensive diseases in 2 (8.7%) and 21 (91.3%) patients, respectively. In addition, the results of the Cohen’s kappa demonstrated a strong (κ=0.82), fair (κ=0.24), and poor (κ=0.12) agreement between the PET/CT and CT findings for determining tumor, node, and metastasis stages, respectively. The ^18^F-FDG PET/CT scans upstaged disease in 47% of the cases with visceral and osseous metastasis. The disease-free survival rates for the limited and extensive diseases were 100% and 23% within the 12-month follow-up. In addition, 8 (35%) patients expired during the follow-up period.

**Conclusion:**

Improved nodal and metastatic disease identification highlights the role of ^18^F-FDG PET/CT scanning in initial staging of SCLC with prognostic implications.

## Introduction

Small cell lung cancer (SCLC) is an aggressive neuroendocrine carcinoma, which accounts for 10-15% of the pulmonary cancers. As a systemic disease, the clinical course and prognosis of SCLC is entirely different from the non-small cell lung cancer (NSCLC) (1). SCLC presents with either limited disease, which is confined to ipsilateral thorax within a single radiation port, or extensive disease having T3-4 with or without distant metastases. In cases the metastatic spread occurs at the early stages of SCLC, the patients are rarely candidates for surgery (2).

There is growing evidence regarding the higher sensitivity of fluorine-*18* fluorodeoxyglucose (*^18^*F-*FDG*) *positron emission tomography*-*computed tomography* (PET/*CT*) scanning in the staging of pulmonary cancer, compared with the conventional imaging to triage the surgical candidates. However, limited data is available for SCLC staging through PET/CT scanning in comparison to the NSCLC since most of these patients are not surgical candidates (3).

With this background in mind, the present study aimed to determine the added value of ^18^F-FDG PET/CT in tumor, node, and metastasis (TNM) staging of SCLC as compared to the conventional CT scan and its potential role as a prognosticator.

## Methods

### Patient population

This retrospective review was conducted on all consecutive patients referred for ^18^F-FDG PET/CT baseline staging within October 2009-December 2015. Based on the histopathological studies, a total of 23 cases were identified to suffer from SCLC. All the patients underwent conventional contrast-enhanced CT of the chest/abdomen and integrated whole-body ^18^F-FDG PET/CT scans. The data set comprised of 19 males and 4 females with the mean age of 58±9 years. The initial conventional contrast-enhanced CT scan of each patient was available for comparison.

### ^18^F FDG PET/CT scanning

All patients underwent ^18^F-FDG PET/CT scanning 60 min after receiving 300 MBq ^18^F-FDG through intravenous injection. Fasting of at least 4 h prior to the injection was assured in all the patients. The image acquisition was performed by a dedicated PET/CT scanner (Phillips Gemini TOF) with 8-9 bed positions (3 min for each position). The CT scan was acquired over 1 min with a voltage of 70-140 kVp and a tube current of 80 mA. Both corrected and uncorrected PET images were evaluated in the visual assessment and *standardized uptake value* (SUV) estimation of metabolic activity.

### Disease staging

The TNM staging was determined using the diagnostic CT and PET/CT scans (4). The disease confined to one hemi-thorax with regional lymph nodes, which could be covered by tolerable radiotherapy fields was categorized as limited disease. On the other hand, the disease beyond single hemi-thorax with or without contralateral adenopathy, pulmonary nodularity, or distant metastasis was labeled as extensive disease.

### Statistical analysis

The data were presented in terms of frequency and percentage, where applicable. The Cohen’s kappa (κ) was applied to determine the degree of agreement between the results of the ^18^F-FDG PET/CT and CT scans for tumor, nodal, and metastatic stage status of SCLS. The Chi-square test was employed to evaluate the significance of difference between the results of the diagnostic CT and ^18^F-FDG PET/CT scans. P-values less than 0.05 was considered statistically significance.

## Results

### Tumor staging

Out of the 23 patients, 3 (13%), 3 (13%), and 17 (74%) cases were staged T2, T3, and T4, respectively. The ^18^F-FDG PET/CT upstaged tumor in three cases. Furthermore, there was a strong agreement between the ^18^F-FDG PET/CT and CT scans for determining the T stage (κ=0.82).

### Nodal staging

Regarding the nodal stages, 3 (13%), 3 (13%), and 17 (74%) patients were identified to be at N0, N2, and N3, respectively. The ^18^F-FDG PET/CT imaging upstaged the nodal disease in 10 patients as compared to the CT scan. The weighted κ values revealed a fair degree of agreement between the PET/CT and CT results (κ=0.24).

### Metastatic staging

The ^18^F-FDG PET/CT scan showed metastatic disease in 15 (65%) patients. In comparison to CT scan, additional metastasis to bone (39%), brain (4%), liver (17%), and adrenal glands (8.7%) were identified on the PET/CT scans. In addition, no agreement was observed between the results of ^18^F-FDG PET/CT and CT scans for M staging (κ=0.12).

### Overall staging

The ^18^F-FDG PET/CT imaging identified TNM stages I (n=2), III (n=6), and IV (n=15). Out of the 23 patients, 21 (91.3%) cases had extensive disease, and 2 (8.7%) subjects had limited disease. The ^18^F-FDG PET/CT scan upstaged disease in 11 (47%) patients with visceral and osseous metastasis, compared to the diagnostic CT scan. Out of these 11 cases, 5 (45%) patients were upstaged from limited disease to extensive disease (P=0.08). The details of TNM staging on diagnostic CT and ^18^F-FDG PET/CT scans are displayed in tables 1 and 2.

**Table 1:** Details of TNM stage on diagnostic CT versus ^18^F FDG PET-CT scan

Sr.No	TNM Diagnostic CT	TNM PET-CT	TNM Upstaged on PET-CT	Disease Status at 12 months’ follow-up
1	T4 N2 M0	T4 N2 M0	No	Alive with disease
2	T3 N3 M0	T3 N3 M0	No	Alive with disease
3	T3 N1 M0	T4 N2 M1b	Yes	Dead
4	T4 N3 M0	T4 N3 M0	No	Alive with disease
5	T3 N1 M0	T4 N3 M1b	Yes	Dead
6	T2 N0 M0	T2 N0 M0	No	Alive and disease free
7	T4 N2 M1b	T4 N3 M1b	No	Alive with disease
8	T2 N0 M0	T2 N0 M0	No	Alive and disease free
9	T4 N3 M0	T4 N3 M1b	Yes	Dead
10	T2 N2 M0	T2 N3 M0	Yes	Alive with disease
11	T3 N0 M0	T3 N2 M1b	Yes	Dead
12	T3 N1 M0	T4 N3 M1b	Yes	Dead
13	T3 N3 M0	T3 N3 M1b	Yes	Lost to follow-up
14	T4 N3 M0	T4 N3 M1b	Yes	Dead
15	T4 N3 M0	T4 N3 M0	No	Alive with disease
16	T4 N3 M0	T4 N3 M0	No	Alive with disease
17	T4 N0 M0	T4 N0 M0	No	Alive with disease
18	T4 N3 M0	T4 N3 M1b	Yes	Dead
19	T4 N2 M1b	T4 N3 M1b	No	Lost to follow-up
20	T4 N3 M0	T4 N3 M0	No	Alive with disease
21	T4 N1 M1a	T4 N3 M1b	Yes	Alive with disease
22	T4 N3 M0	T4 N3 M1b	Yes	Lost to follow-up
23	T4 N1 M0	T4 N3 M1b	Yes	Dead

**Table 2:** Disease extent - CT versus ^18^F FDG PET-CT scan (P=0.08)

N = 23	CT	^18^F FDG PET-CT
Limited Disease	7(30%)	2(8.7%)
Extensive Disease	16(70%)	21(91.3%)

Based on the disease stage, 3, 7, and 13 patients underwent surgical resection, chemotherapy followed by radiotherapy, palliative chemotherapy respectively. Furthermore, the management of five patients were changed from curative to palliative treatment.

### Survival analysis on the basis of PET/CT staging

The median follow-up time was eight months, ranging 3-27 months. The follow-up was based on the clinical and imaging data. The duration of overall survival was calculated from the time of staging ^18^F-FDG PET/CT scan. Both of the two patients with limited disease were alive and disease-free during the follow-up period.

**Figure 1:**
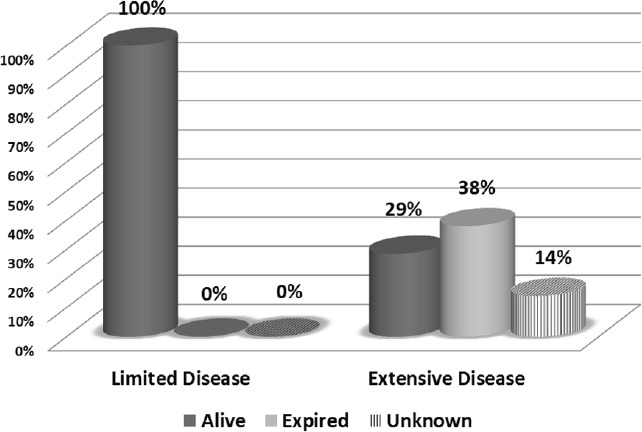
Overall survival status based on the disease extent on FDG PET/CT scans

In the group with extensive disease, 6 (28.5%) patients were alive with persistent or progressive disease, and 8 (38%) patients expired during the follow-up period. None of these patients achieved disease-free status. Furthermore, three patients were lost to follow-up. Figure 1 displays the overall disease status at the end of the follow-up period.

## Discussion

Accurate staging is the mainstay of the SCLC management. The main aim of the staging workup is to triage the patients who would benefit from surgery. The limited-stage SCLC is considered to be potentially curable, rendering adequate and accurate staging, which is necessary for surgical decision and therapy planning. SCLC commonly metastasizes to bones, liver, brain, and adrenal glands. The standard imaging workup comprises of CT and magnetic resonance imaging scanning (5).

Hybrid ^18^F-FDG PET/CT scan, carrying dual information regarding the metabolic and anatomic status of disease, has been widely incorporated in staging workup for NSCLC lately. However, limited data is available when taking the SCLC in to account. In a meta-analysis conducted by Kalemkerian et al. in 2013, a total of 14 studies were reported evaluating and comparing the pretreatment ^18^F-FDG PET/CT scanning with conventional imaging. None of the reported literature had a larger SCLC cohort (5-7). Given the limited literature and considering the lack of the related studies in Pakistan, this review was conducted to study the added value or prognostication of PET/CT scan in the oncological referrals in this country.

The ^18^F-FDG PET/CT was revealed to have a sensitivity of 100% in tumor staging. The SCLC has a high metabolic activity, taking up FDG radiotracer with high standardized uptake values. This makes the PET/CT scan highly sensitive in terms of the primary tumor localization and determination of its accurate extent (8). In our data set, ^18^F-FDG PET/CT imaging was found to be better in nodal staging, compared to the conventional CT scanning, which is in line with the literature.

Accurate nodal staging is critical in deciding about the surgical resection of lung. Since nodal status on CT scan is based on the morphology, any node smaller than a centimeter in diameter is disregarded. FDG PET/CT scan has higher diagnostic accuracy in identifying disease in smaller nodes with higher metabolic status since the metabolic changes are known to precede the morphological changes (9, 10). This is applicable to both NSCLC and SCLC.

The present study revealed the superiority of integrated ^18^F-FDG PET/CT scanning over the CT imaging in the detection of visceral and osseous metastases. Additional metastases was detected in 11 patients on the PET/CT scans, compared to the conventional CT images (κ=0.12). Likewise, Brink et al. reported higher sensitivity and specificity of ^18^F-FDG PET/CT scanning when screening for metastatic spread in the SCLC, except for the brain metastases (11). Nevertheless, Fischer et al. showed the 93 % sensitivity and 100% specificity of PET/CT scan in detecting extensive SCLC disease (12).

Out of the 23 patients, upstaged disease in 11 patients led to a change in the management plan with alteration in the general treatment (n=7) or surgical decision (n=5). Kalemkerian et al. has reviewed several studies on the change of SCLC staging based on PET/CT scanning. They reported that the PET/CT findings led to a change in initial management in 28% (range=0-47%) of the patients (5).

We studied the prognostic role of ^18^F-FDG PET/CT scanning in the SCLC. Although we had only two patients with limited disease on baseline scan, the overall survival of limited disease was better than the extensive one. Since the PET/CT scan facilitated the accurate staging of SCLC, the patients with limited disease benefitted from surgical resection and had a disease-free survival of 100% in the 12-month follow-up. However, this rate was 23% in the patients with extensive disease. Azad et al. reported a longer overall survival (18.6 months) in the limited disease, compared to that (5.7 months) in the extensive disease (P<0.0001) (13).

In summary, SCLC is an FDG-avid disease; however, due to the aggressive nature of this disease, the clinical data on validating the use of PET scan in the baseline workup of the patients with this disease is limited, compared to that in the NSCLC. ^18^F-FDG PET/CT scan can accurately distinguish between the limited and extensive diseases of SCLC. Since PET/CT scanning is less frequently used in the staging of SCLC, a potential curable, but smaller proportion of patients can be missed. Staging ^18^F-FDG PET/CT imaging has a favorable impact on the respective outcome as a prognosticator.

The major limitation of our study was the small sample size and limited follow-up period, which was primarily due to our institutional triage policy.

## Conclusion

^18^F-FDG PET/CT scanning improves the identification of nodal and metastatic disease in SCLC. The accurate evaluation of the potentially curable limited versus extensive disease highlights the role of PET/CT in initial staging of SCLC. With appropriately directed management plan, there is prognostic implication of FDG PET/CT scan as well.

## Conflicts of interest

The authors reported no conflicts of interest.
